# Secretory glycoprotein NS1 plays a crucial role in the particle formation of flaviviruses

**DOI:** 10.1371/journal.ppat.1010593

**Published:** 2022-06-03

**Authors:** Tomokazu Tamura, Shiho Torii, Kentaro Kajiwara, Itsuki Anzai, Yoichiro Fujioka, Kisho Noda, Shuhei Taguwa, Yuhei Morioka, Rigel Suzuki, Yuzy Fauzyah, Chikako Ono, Yusuke Ohba, Masato Okada, Takasuke Fukuhara, Yoshiharu Matsuura

**Affiliations:** 1 Department of Molecular Virology, Research Institute for Microbial Diseases, Osaka University, Suita, Osaka, Japan; 2 Department of Microbiology and Immunology, Faculty of Medicine, Hokkaido University, Sapporo, Hokkaido, Japan; 3 Laboratory of Virus Control, Center for Infectious Disease Education and Research, Osaka University, Suita, Osaka, Japan; 4 Department of Oncogene Research, Research Institute for Microbial Diseases, Osaka University, Suita, Osaka, Japan; 5 Department of Cell Physiology, Faculty of Medicine, Hokkaido University, Sapporo, Hokkaido, Japan; 6 Global Station for Biosurfaces and Drug Discovery, Hokkaido University, Sapporo, Hokkaido, Japan; 7 AMED-CREST, Japan Agency for Medical Research and Development, Sapporo, Hokkaido, Japan; Duke University Medical Center, UNITED STATES

## Abstract

Flaviviruses, which are globally distributed and cause a spectrum of potentially severe illnesses, pose a major threat to public health. Although *Flaviviridae* viruses, including flaviviruses, possess similar genome structures, only the flaviviruses encode the non-structural protein NS1, which resides in the endoplasmic reticulum (ER) and is secreted from cells after oligomerization. The ER-resident NS1 is known to be involved in viral genome replication, but the essential roles of secretory NS1 in the virus life cycle are not fully understood. Here we characterized the roles of secretory NS1 in the particle formation of flaviviruses. We first identified an amino acid residue essential for the NS1 secretion but not for viral genome replication by using protein-protein interaction network analyses and mutagenesis scanning. By using the recombinant flaviviruses carrying the identified NS1 mutation, we clarified that the mutant flaviviruses employed viral genome replication. We then constructed a recombinant NS1 with the identified mutation and demonstrated by physicochemical assays that the mutant NS1 was unable to form a proper oligomer or associate with liposomes. Finally, we showed that the functions of NS1 that were lost by the identified mutation could be compensated for by the in trans-expression of E^rns^ of pestiviruses and host exchangeable apolipoproteins, which participate in the infectious particle formation of pestiviruses and hepaciviruses in the family *Flaviviridae*, respectively. Collectively, our study suggests that secretory NS1 plays a role in the particle formation of flaviviruses through its interaction with the lipid membrane.

## Author summary

It is difficult to characterize the function of NS1 in the post-genome replication stages in the virus life cycle of flaviviruses. Here, by means of protein-protein interaction network analyses and mutagenesis scanning, we identified a unique mutation in NS1 by which the protein loses its secretory capacity while retaining its genome replication activity. Physicochemical assays using the mutant NS1 revealed that oligomerization of NS1 is responsible for the lipid association and secretion of NS1. In addition, we established a complementation assay that can evaluate the particle formation of *Flaviviridae* viruses. By using recombinant flaviviruses possessing the identified mutation in NS1, we clarified that NS1 is involved in particle formation. Our findings reveal that the flavivirus NS1 has at least two roles in the virus life cycles—namely, a role in infectious particle formation and a role in viral genome replication.

## Introduction

The family *Flaviviridae* viruses consist of 4 genera: *Flavivirus*, *Pestivirus*, *Pegivirus* and *Hepacivirus* [1]. Although flaviviruses and pestiviruses can infect various species and tissues, infection of pegiviruses and hepaciviruses is observed in a strikingly restricted range of tissues and hosts. Flaviviruses can be readily transmitted by arthropod vectors to a variety of mammalian species and (re-) emerge unexpectedly in human populations [2]. Some of the mosquito-transmitted flaviviruses, including dengue virus (DENV), Japanese encephalitis virus (JEV), West Nile virus (WNV), yellow fever virus (YFV), and Zika virus (ZIKV), have expanded globally and cause febrile illness to humans. The limited availability of vaccines or specific therapeutics for the mosquito-borne flaviviruses continues to pose a threat to public health. Therefore, an improved understanding of the molecular mechanisms underlying the life cycle of these viruses is needed for the development of effective countermeasures.

*Flaviviridae* viruses share a common genome structure consisting of a single-stranded and positive-sense RNA with one open reading frame [1]. Interestingly, flaviviruses possess a unique non-structural protein within the *Flaviviridae* family. This protein is NS1, a 48- to 55-kD glycoprotein that is well-conserved among flaviviruses, exhibiting 20%–40% identity and 60%–80% similarity at the amino acid level [3, 4]. NS1 is initially synthesized as a soluble monomer and becomes membrane-associated after dimerization in the lumen of the endoplasmic reticulum (ER). The higher oligomeric NS1 is secreted into extracellular environments. While the dimer of NS1 is involved in the formation of a replication complex in the ER, the secretory NS1 is responsible for dengue pathogenesis via induction of the release of vasoactive cytokines [5]. The secretory NS1 also plays a role in immune evasion by binding the complement protein C4 and triggering its degradation [6]. In addition, NS1 can directly alter the barrier function of pulmonary endothelial cell monolayers through disruption of the tissue-specific endothelial glycocalyx-like layer by triggering the activation of endothelial sialidases *in vitro* and *in vivo* [7, 8]. Because DENV NS1 circulates at high levels (1–2 μg/ml) during the acute phase of infection with severe dengue disease, which correlates with viremia [9], the secretory NS1 is utilized as a marker for the diagnosis of infection [10]. Collectively, these findings demonstrate that the secretory NS1 plays multifunctional roles in the pathogenesis and tissue-specificity of flaviviruses; however, the roles of secretory NS1 in the virus life cycle have not yet been fully characterized.

In our previous studies, the amphipathic α-helices of exchangeable apolipoproteins were shown to play crucial roles in the formation of infectious particles of hepaciviruses through their interaction with viral particles [11]. We further showed that pestivirus E^rns^ and flavivirus NS1 play functional roles similar to those of host-derived apolipoproteins in the formation of infectious particles of hepaciviruses and pestiviruses [12]. These results suggest that the host- and virus-derived secretory glycoproteins have overlapping roles in the virus life cycle of *Flaviviridae* viruses, especially in the maturation of infectious particles. We therefore hypothesized that NS1 plays crucial roles in the maturation of infectious particles of flaviviruses.

Here, we investigated the functional roles of secretory NS1 in the flavivirus life cycle. We showed that a loss-of-function mutant of NS1 lacking secretory ability was substantially involved in replication of viral RNA but failed to interact with the lipid membrane or to produce infectious particles. Moreover, the virus carrying the mutant NS1 could restore the particle production by the expression of the lipid associated NS1.These findings suggest that NS1 plays at least two roles in the virus life cycle. In addition, we proved that the pestivirus E^rns^ and host-derived exchangeable apolipoproteins can compensate for the loss of function of secretory NS1 in the flavivirus particle formation. Taken together, the results of this study shed light on the functions of flavivirus NS1 in the virus life cycle.

## Results

### Generation of a reporter ZIKV that is unable to replicate but is rescued by the exogenous expression of NS1

**Fig 1:**
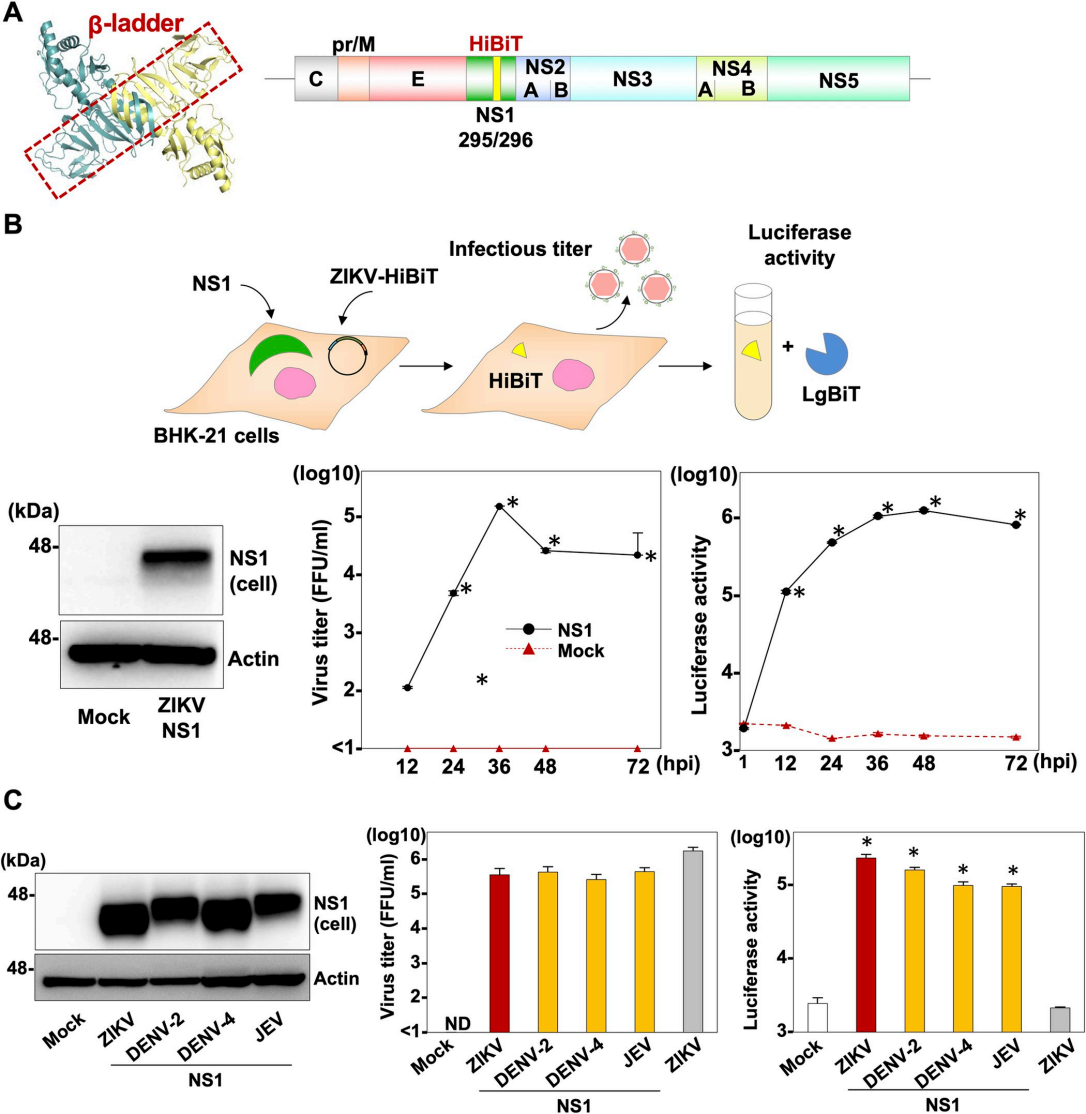
Generation of a reporter ZIKV that is unable to replicate but is rescued by the exogenous expression of NS1. (A) The HiBiT luciferase was inserted in the β-ladder of NS1 (NS1 dimer: Protein Data Bank accession number: 4TPL). A schematic representation of the recombinant ZIKV carrying the HiBiT luciferase in the NS1 gene (ZIKV-HiBiT). The HiBiT luciferase sequence with the GS linker was inserted at position 295 aa of NS1. (B) An illustration shows the experimental workflow. Expression of the ZIKV NS1 protein was determined by immunoblotting at 48 hpi of lentiviruses into BHK-21 cells. Luciferase activity in cells and infectious titers in the culture supernatants were determined upon infection of ZIKV-HiBiT at MOI = 0.1 into either parental cells (Mock) or cells expressing ZIKV NS1 (NS1) at the indicated timepoints. (C) NS1 proteins from ZIKV, DENV-2, DENV-4, and JEV were expressed in BHK-21 cells. Luciferase activities in the cells and infectious titers in the culture supernatants were determined at 36 hpt of ZIKV-HiBiT into cells expressing these NS1proteins. Statistical significance was assessed using Student’s *t*-test (B) or one-way ANOVA with Dunnett’s test (C) and is indicated by asterisks (*) (versus control cells).

First, to investigate the role of NS1 in virus propagation, we generated cDNA clones having a deletion in the NS1 gene of flaviviruses JEV and DENV. We tried to conduct a complementation assay by transfecting the *in vitro*-transcribed RNAs into Vero E6 cells expressing the NS1 protein (S1A Fig). We collected the supernatants of the transfected cells at 96 h post-transfection (hpt) and measured infectious titers. However, no infectious virus was recovered, suggesting that trans-complementation of NS1 cannot be achieved by using cDNA clones possessing deletion in the NS1 genes of JEV and DENV (S1B and S1C Fig). We therefore took the alternative approach of inserting the high-affinity NanoBiT (HiBiT) luciferase gene [13, 14] into the β-ladder region of NS1 (Fig 1A). The HiBiT luciferase is a split luciferase gene encoding 11 amino acids and is one of the components of the NanoBiT. When HiBiT and its counterpart large NanoBiT (LgBiT) associate in cells or *in vitro*, the complex regains its NanoLuc enzymatic activity, which correlates with viral replication of ZIKV. The resulting cDNA was cloned into a vector under the control of a CMV promoter and hepatitis delta virus ribozyme cassette (ZIKV-HiBiT) and transfected into BHK-21 cells (Fig 1B). Although no RNA replication was observed in the BHK-21 cells (Mock in Fig 1B), both RNA replication and infectious particles were rescued by the expression of wild type NS1 of ZIKV (NS1 in Fig 1B). In addition, when we similarly expressed the NS1 protein of other flaviviruses, including DENV and JEV, in the BHK-21 cells, infectious particles were recovered in the supernatants of the ZIKV-HiBiT clone-transfected cells at levels comparable to those from the wild type ZIKV infection (Fig 1C, middle), suggesting that the loss of the functional role of NS1 of ZIKV can be compensated for by the expression of NS1 derived from other flaviviruses. Moreover, significant luciferase activity was observed in the culture supernatants of cells transfected with the ZIKV-HiBiT clone by the exogenous expression of NS1 of flaviviruses (Fig 1C, right), suggesting the possibility of identifying the amino acid residue(s) in NS1 that is crucial for RNA replication and infectious particle formation by measuring luciferase activities.

### Identification of an amino acid residue involved in higher oligomerization of NS1 through *in silico* and mutagenesis analyses

**Fig 2:**
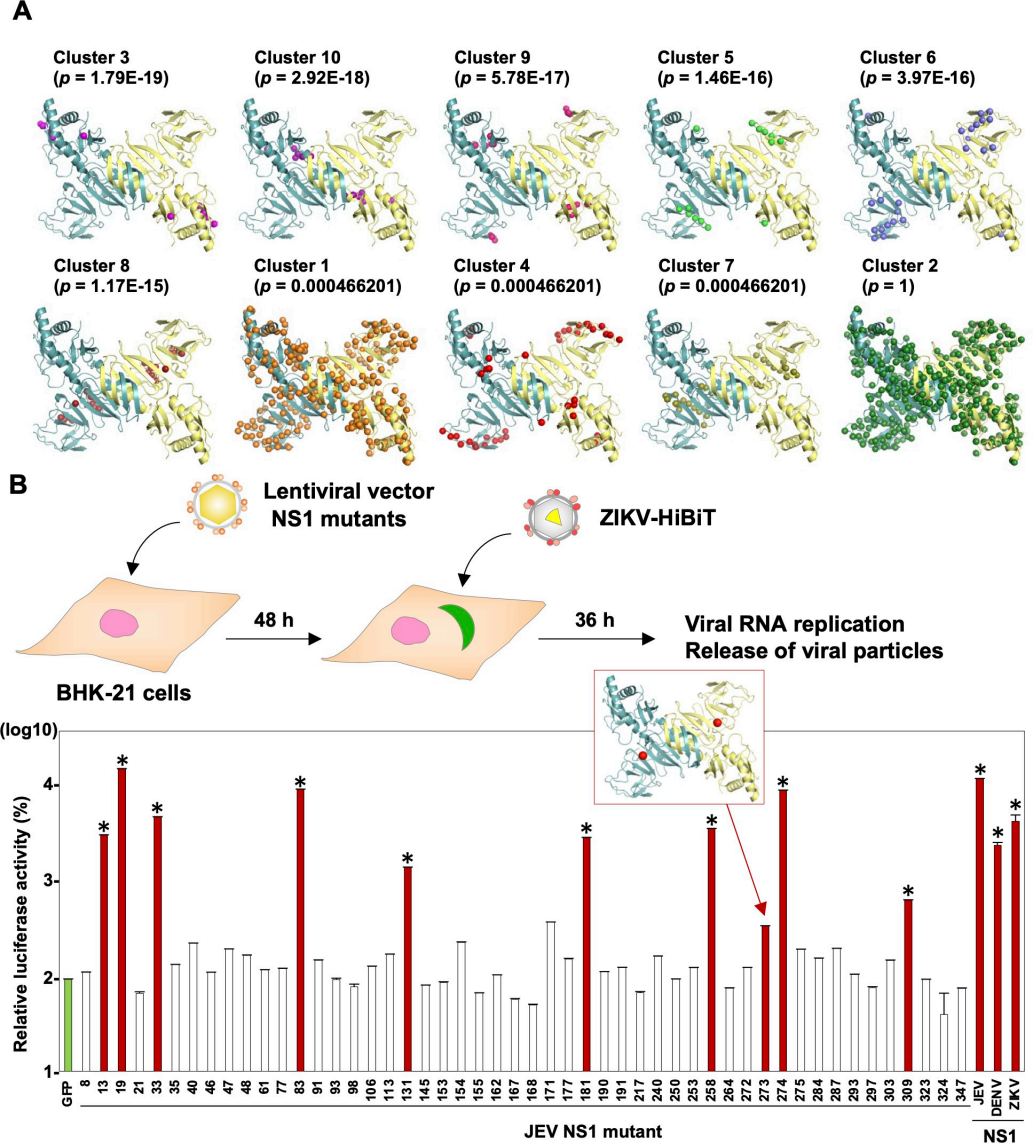
Identification of an amino acid residue necessary for the secretion of NS1 through *in silico* and mutagenesis analyses. (A) The protein-protein network within the NS1 protein was analyzed by using co-evolving amino-acid pairs [16, 17] with 66 strains belonging to the Japanese encephalitis serocomplex group. The amino acid residues possessing interaction within clusters are shown as spheres in the three-dimensional structures of NS1 dimers (Protein Data Bank accession number: 4TPL). (B) An illustration shows the experimental workflow. Upon infection with ZIKV-HiBiT at MOI = 0.1, the luciferase activity of cells expressing NS1 mutants carrying the amino acids with different phenotypes (S2 Table) were measured at 36 hpi. The relative luciferase activity was normalized by the activity of cells expressing GFP. The production of double-stranded RNA (dsRNA) was determined by staining with antibody against dsRNA and is indicated by red bars. The locus of amino acid position 273 on the NS1 structure is indicated as red spheres. The data set of the quantified fluorescent signal of dsRNA are found in S2B Fig. Statistical significance was assessed using one-way ANOVA with Dunnett’s test (B) and is indicated by asterisks (*) (versus control cells).

Having established a complementation assays for evaluating the roles of NS1 in the virus life cycle, we next attempted to conduct mutagenesis scanning using ZIKV-HiBiT. NS1 of flaviviruses has been reported to form a dimer and higher oligomer in the infected cells. The dimer is considered to participate in the viral genome replication and the higher oligomer is secreted into the culture supernatants [15]. With the aim of identifying amino acid residue(s) involved in the secretory form of NS1, we utilized a computational coevolution-based method, Blocks in Sequences (BIS) [16, 17]. BIS is able to calculate the protein-protein interaction network within a protein. Based on the BIS analyses of 66 different strains of Japanese encephalitis serocomplex, 10 clusters were identified (Fig 2A and S1 Table) and 9 clusters (clusters 1, 3–10) exhibited different *p*-values ranging between 4 x 10^−4^ and 2 x 10^−19^, and cluster 2 was conserved (*p*-value = 1). To investigate which clusters or amino acid residues are involved in the formation of the higher oligomer, we changed the amino acid at each position to an amino acid with characteristics (S2 Table) in terms of destroying or altering NS1 oligomer formation. In total, 50 BHK-21 cells expressing the respective NS1 mutants were generated (S2A Fig) and subjected to our complementation assay. Cells expressing the respective NS1 mutants were infected with ZIKV-HiBiT, and the luciferase activity of the supernatants was measured at 96 h post-infection (hpi). In addition, we fixed the infected cells and used an immunofluorescence assay to detect dsRNA as an index of active RNA replication (Figs 2B and S2B). dsRNA signals were detected upon infection with ZIKV-HiBiT in 10 of the 50 NS1 mutant-expressing cells (indicated by red bars; Fig 2B and by red bots; S2B Fig), indicating that these NS1 mutants were able to support viral replication *in trans*. Among the 10 NS1 mutants possessing replication activity, the NS1 carrying histidine instead of isoleucine at position 273 (NS1_I273H_) exhibited luciferase activity but at significantly lower levels compared to the wild type NS1 (Fig 2B), as represented by the red dots in cluster 8 (shown in the red square in Fig 2B). Also, an active RNA replication was impaired by this substitution (S2B Fig). Collectively, these data suggest that the mutant NS1_I273H_ remains a dimer but might have lost the ability to form the higher oligomer.

### Basic amino acid residues at position 273 of NS1 play a crucial role in secretion

**Fig 3:**
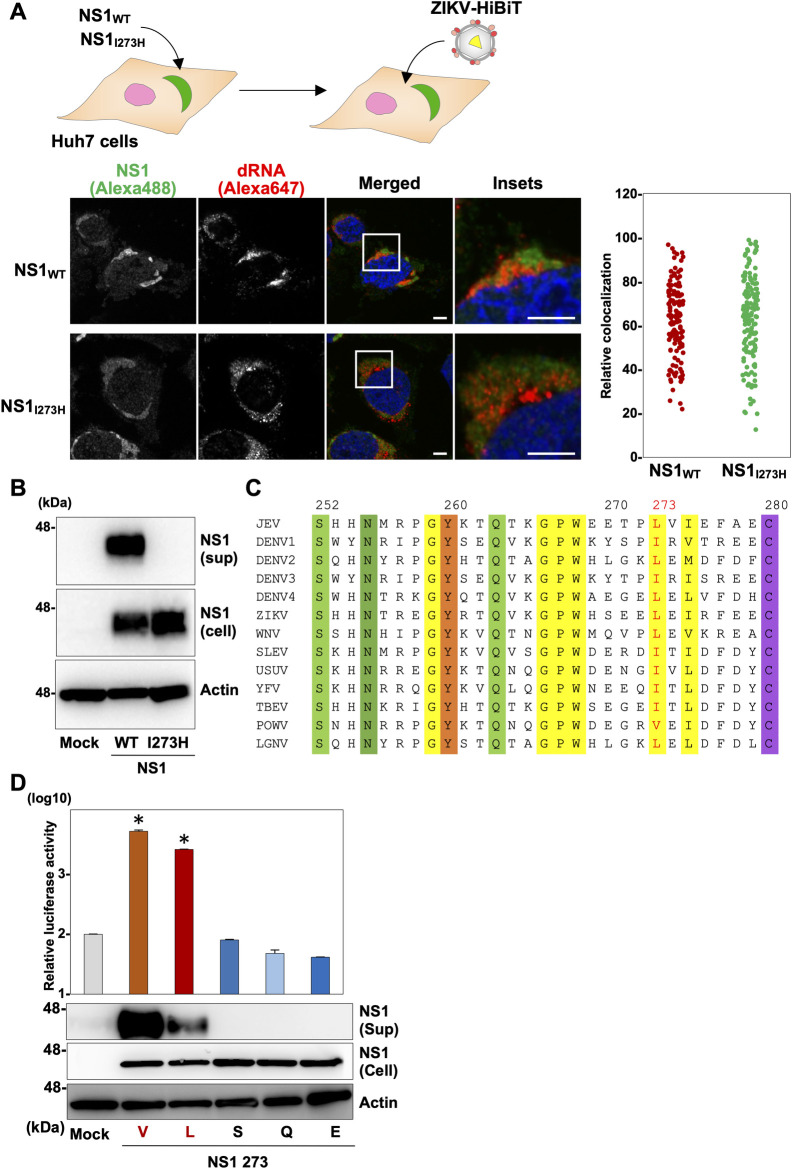
Basic amino acids residues at position 273 of NS1 play a crucial role in the secretion of NS1. (A) An illustration shows the experimental workflow. Either the wild type or I273H mutant NS1 was expressed in Huh7 cells. Localization of NS1 and dsRNA in cells infected with ZIKV-HiBiT was observed at 48 hpi by confocal microscopy using antibodies against NS1 (red dots in the left panels) and dsRNA (green dots in the middle panels), respectively. The nuclei were counterstaining by Hoechest 33342. The white bar represents 10 μm. dsRNA- and NS1-positive regions were extracted and their colocalization was determined and shown as a dot graph. (B) Intracellular (Cell) and extracellular (Sup) expressions of the wild type NS1 and NS1_I273H_ mutant were determined by immunoblotting. (C) The amino acid (252–280 aa of NS1) alignments of the 13 viruses belonging to the *Flavivirus* genus are shown. (D) Relative luciferase activities of the culture supernatants of BHK-21 cells expressing the wild type and mutant NS1 substituted at position 273 (valine, leucine, serine, glutamine, and glutamic acid) upon infection with the ZIKV HiBiT were determined. Secretion of NS1 into the supernatants was determined by immunoblotting. Statistical significance was assessed using one-way ANOVA with Dunnett’s test (D) and is indicated by asterisks (*) (versus control cells).

Next, Huh7 cells expressing the wild type or mutant (NS1_I273H_) of JEV were infected with ZIKV-HiBiT and the synthesis of dsRNA and expression of NS1 were examined at 48 hpi by confocal microscopy (Fig 3A). The immunofluorescent signals for NS1 (green) and dsRNA (red) were observed in the cytoplasm of both cells, and some of them were colocalized. Next, secretion of NS1 was detected by immunoblotting of culture supernatants of Huh7 cells expressing either the wild type or mutant NS1 of JEV collected at 96 hpt. Although both NS1 proteins were expressed in cells, the wild type NS1 but not the NS1_I273H_ mutant was secreted into the culture supernatants (Fig 3B). These results suggest that the isoleucine residue at position 273 in NS1 plays a crucial role in the secretion of JEV NS1. In addition, we found that the property of the amino acid at position 273 of NS1 in JEV is conserved among flaviviruses (Fig 3C). To investigate the effect of amino acid substitution at this position, we conducted a complementation experiment as shown in Fig 2B using NS1 possessing an amino acid mutation at position 273. NS1 mutants carrying an amino acid at position 273 with the same property as isoleucine (valine or leucine) were secreted into the supernatants, but those with a different property (serine, glutamine, or glutamic acid) were not (Fig 3D), suggesting that the basic amino acids residues at position 273 play a crucial role in the secretion of NS1 and the subsequent infectious particle production.

### The non-secretory NS1_I273H_ sustains the role of RNA replication and is compensated the role of the infectious particle formation by the replication-defective NS1_F160D_

**Fig 4:**
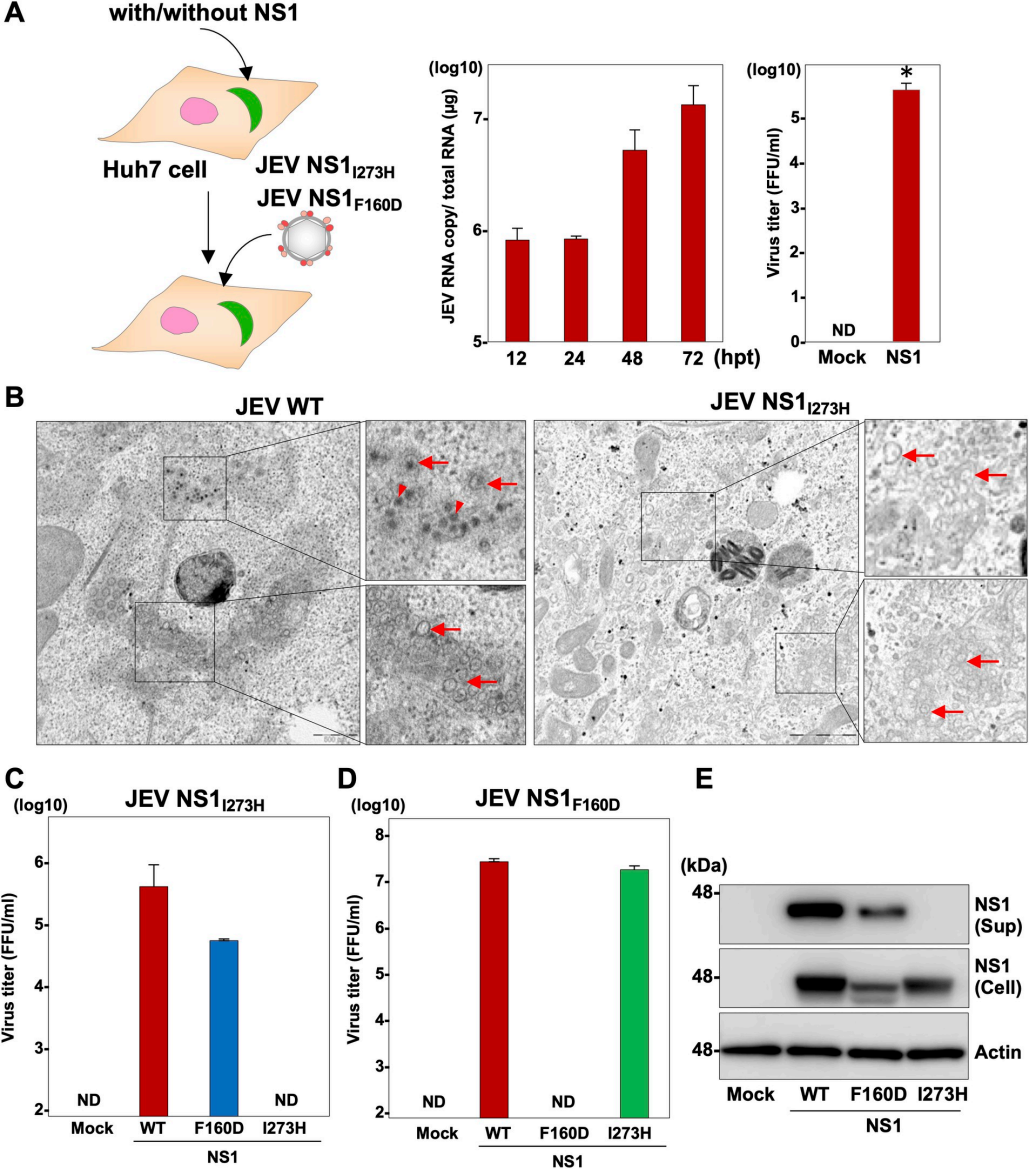
The non-secretory NS1_I273H_ sustains the role of RNA replication and is compensated for the role of the infectious particle formation by the replication-defective NS1_F160D_. (A) An illustration shows the experimental workflow. JEV NS1_I273H_ was infected into Huh7 cells and RNA copies were determined at 12, 24, 48, and 72 hpi. Recovery of infectious particles in the culture supernatants of Huh7 cells expressing JEV NS1 (NS1) upon infection with JEV NS1_I273H_. (B) Huh7 cells infected with JEV (left) or JEV NS1_I273H_ (right) were fixed at 48 hpi and observed by transmission electron microscopy. Arrowheads and arrows indicate electron-dense virus particles and double membrane-derived vesicles, respectively. Huh7 cells or Huh7 cells expressing either the wild type, F160D, or I273H were infected with JEV NS1_I273H_ (C) or JEV NS1_F160D_ (D) and infectious titers were measured at 96 hpi. (E) Expressions of the wild type and mutant NS1 protein in the culture supernatants (Sup) and cells (Cell) were determined by immunoblotting at 48 hpi of lentiviruses into BHK-21 cells. Statistical significance was assessed using Student’s *t*-test (right panel of A) and is indicated by asterisks (*) (versus control cells).

To further characterize the NS1 mutant (NS1_I273H_), we generated a mutant JEV RNA carrying the histidine substitution in NS1 (JEV NS1_I273H_), because we were unable to obtain *in vitro* transcribed RNA from ZIKV possessing the NS1_I273H_ mutation. The JEV mutant RNA was transfected into Huh7 cells, and the replication of viral RNA was determined by RT-qPCR (Fig 4A, left). Substantial viral RNA replication was observed in Huh7 cells from 24 hpt without expressing NS1, suggesting that JEV NS1_I273H_ is capable of replicating in cells. Upon transfection of the JEV NS1_I273H_ RNA, the recovery of infectious particles was only observed in BHK-21 cells at 96 hpi by the expression of NS1, suggesting that I273H substitution kept the role of RNA replication but led to a loss of the capability for infectious virus production (Fig 4A, right). The genome replication of flaviviruses occurs in the intracellular membrane compartments known as replication complexes [18]. To investigate the formation of replication complexes, cells infected with either the wild type JEV or JEV NS1_I273H_ were analyzed by electron microscopy at 48 hpi. Representative images are shown in Fig 4B. Replication complexes consisting of spherules were observed in the cytoplasm of Huh7 cells infected with either the wild type or mutant JEV (denoted by red arrows in Fig 4B). Viral particles were observed in cells infected with the wild type JEV (denoted by red arrowheads in Fig 4B), but not in those infected with JEV NS1_I273H_. These data suggest that JEV NS1_I273H_ is capable of replicating via the formation of RNA replication complexes but is incapable of producing viral particles. Next, a mutant JEV RNA carrying aspartic acid at position 160 (JEV NS1_F160D_) was generated and served as a replication-dead control, because it has been demonstrated that substitution of F160D in the hydrophobic protrusion of DENV NS1 abolishes viral RNA replication [15]. We examined whether the expression of NS1_F160D_ can compensate for the infectious virus production lost in JEV NS1_I273H_ (Fig 4C). Upon infection with the JEV NS1_I273H_, infectious particles were recovered in the supernatants of the cells expressing wild type NS1 and NS1_F160D_ but not in those expressing NS1_I273H_ at 96 hpi, indicating that NS1_F160D_ retains the particle-production function lost in the NS1_I273H_ mutant. When we generated a mutant JEV NS1_F160D_, infectious particles were recovered at 96 hpi in the supernatants of cells expressing the wild type NS1 or NS1_I273H_, but not in those expressing NS1_F160D_ upon infection with JEV NS1_F160D_ (Fig 4D). These results suggest that flavivirus NS1 has at least dual functions in the virus life cycle—i.e., a viral RNA replication function and a particle formation function. In support of this conclusion, we confirmed that NS1 is not involved in viral RNA translation (S3C Fig), and NS1_F160D_ but not NS1_I273H_ was secreted into the culture supernatants of cells expressing these proteins (Fig 4E).

### Higher oligomerization of NS1 is responsible for the membrane association and secretion into culture supernatants

**Fig 5:**
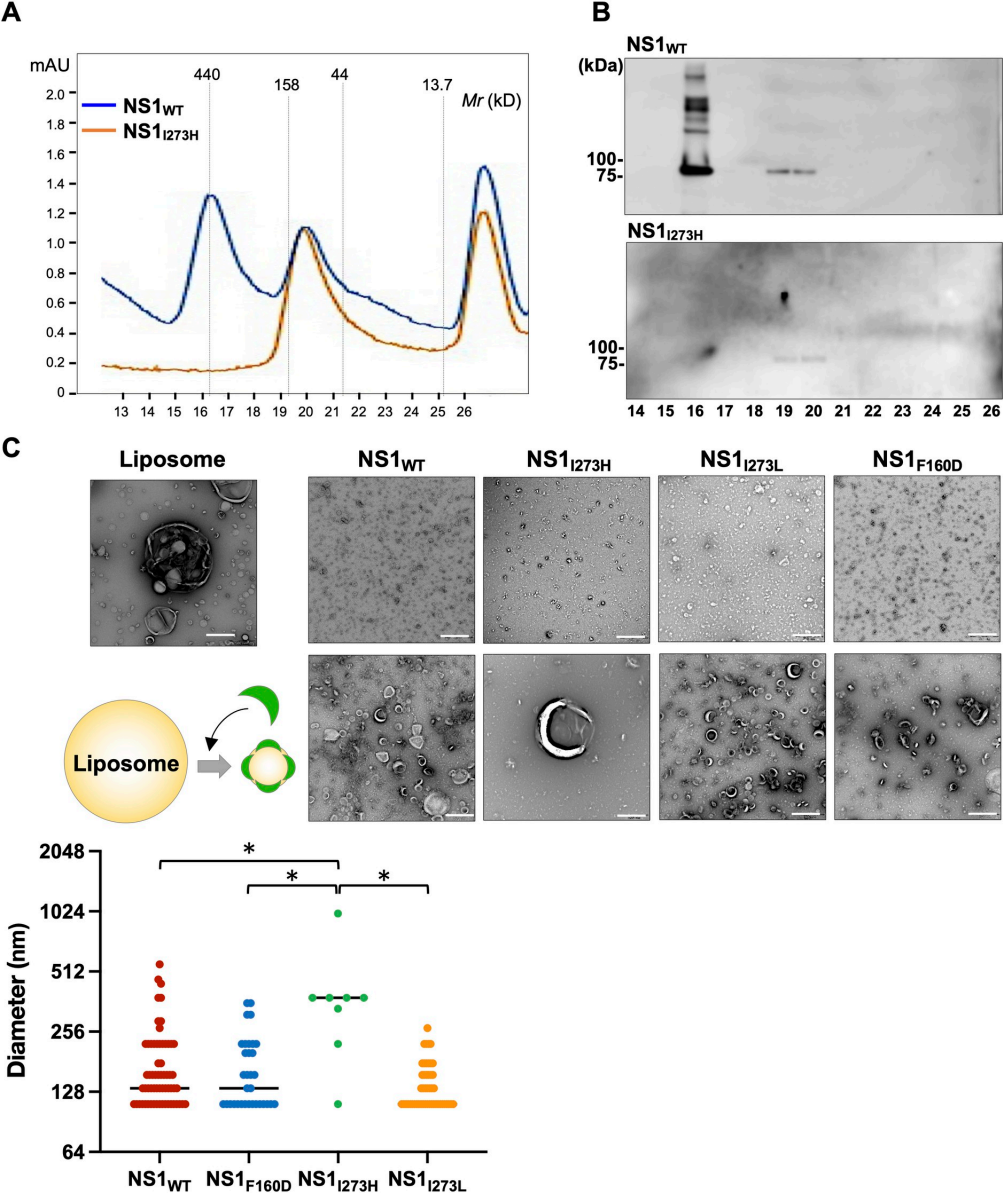
Higher oligomerization of NS1 is responsible for the membrane association and secretion into the culture supernatants. (A) The properties of the wild type NS1 and mutant NS1_I273H_ carrying MBP-tag were analyzed by fast protein liquid chromatography (FPLC). (B) The expressions of the wild type NS1 and mutant NS1_I273H_ in each fraction were confirmed by immunoblotting using anti-NS1 antibody. (C) An illustration shows the image of liposome alteration by incubation with NS1. Conversion of liposomes into small heterogeneous lipid particles was examined by the incubation with NS1. The purified wild type NS1 or mutant NS1 (I273H, I273L and F160D) was incubated with the liposomes (composition 1:9 cholesterol:phosphatidylcholine) and visualized by the negative-stain EM. White scale bars represent 500 nm. The dot graph showed the size of liposome incubated with the respective NS1 proteins. Statistical significances were determined by the Kruskal–Wallis test with pairwise comparisons using Tukey-Kramer-Nemenyi all-pairs test with Tukey-Dist approximation. Significantly different values are indicated by asterisks (*).

Since the BIS analyses showed that the amino acid residue at position 273 of NS1 is involved in protein-protein interaction, we hypothesized that the amino acid residue at 273 participates in the correct oligomer formation of NS1. Thus, we synthesized recombinant MBP-tagged NS1 proteins (approximately 90 kDa) and subjected them to fast protein liquid chromatography (FPLC) to analyze their oligomerization properties (Fig 5A). The peak around 150 kDa (fractions 19 and 20) was observed in both the wild type NS1 and NS1_I273H_ mutant samples, whereas the peak around 300 kDa (fraction 16) was observed only in the wild type NS1. The expression of MBP-NS1 in each fraction was confirmed by immunoblotting using anti-NS1 antibody (Fig 5B). These data suggest that the amino acid residue at position 273 in NS1 participates in higher oligomerization but not dimerization of NS1. In addition, to examine the effect of mutation in NS1_I273H_ on the interaction of NS1 with membranes, the purified NS1 proteins were incubated with liposomes. Conversion of large liposomes (more than 500 nm in diameter) into small heterogeneous lipid particles was observed by the incubation with wild type NS1 and NS1_F160D_ as previously reported [15] (Fig 5C). In addition, NS1_I273L_, which was secreted into culture supernatants (Fig 3D), induced conversion of large liposomes into small lipid particles, but NS1_I273H_ did not alter the liposome morphologies (Fig 5C). These results suggest that the higher oligomerization of NS1 is responsible for the lipid association and secretion into culture supernatants.

### The secretory glycoproteins, including exchangeable apolipoproteins, pestivirus E^rns^, and flavivirus NS1, play a common role in the infectious particle formation of *Flaviviridae* viruses

**Fig 6:**
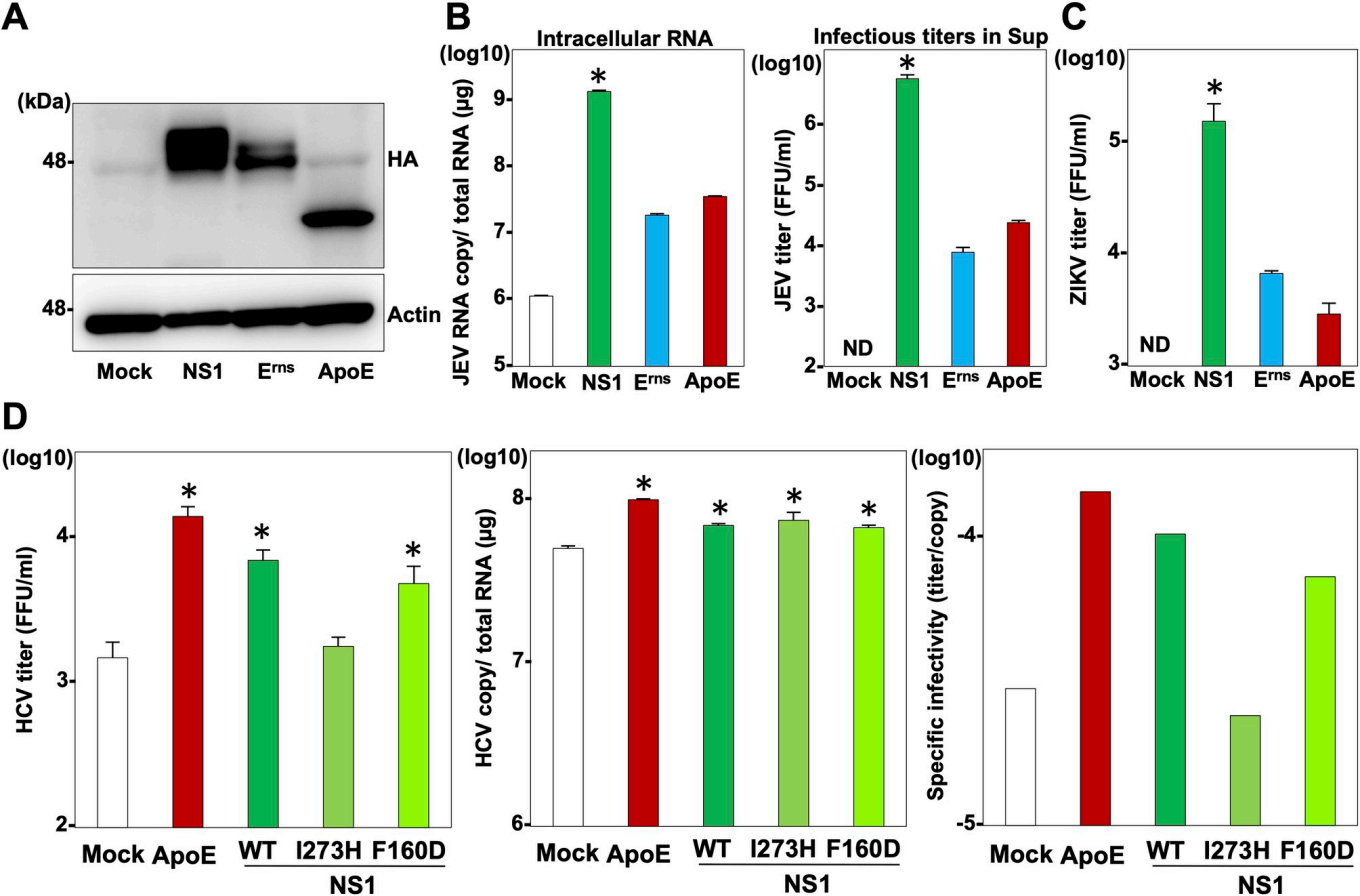
The secretory glycoproteins, including exchangeable apolipoproteins, pestivirus E^rns^, and flavivirus NS1, have a common role in the infectious particle formation of *Flaviviridae* viruses. (A) Expressions of NS1, E^rns^ and ApoE were determined by immunoblotting at 48 hpi of lentiviruses into the BHK-21 cells. (B) At 96 hpi with JEV NS1_I273H_, intracellular JEV RNA and infectious titers in the supernatants were determined by qRT-PCR and focus-forming assay, respectively. (C) At 96 h after infection with ZIKV NS1_I273H_, infectious titers in the supernatants were determined by focus-forming assay. (D) ApoB and ApoE double-knockout Huh7 cells expressing either ApoE, wild type NS1or mutant NS1 (I273H and F160D) were infected with HCV. Infectious titers, intracellular RNA copies and specific infectivity were determined at 3 days post-infection. Statistical significance was assessed using one-way ANOVA with Dunnett’s test (B, C, D) and is indicated by asterisks (*) (versus control cells).

Previously, we have shown that host- and virus-derived secretory glycoproteins, including exchangeable apolipoproteins, pestivirus E^rns^ and flavivirus NS1, play comparable roles in the particle formation of hepaciviruses and pestiviruses in the *Flaviviridae* family [12]. Therefore, we hypothesized that these heterogenous secretory glycoproteins might play common roles in the particle formation of flaviviruses. To examine this possibility, HA-tagged NS1of JEV, pestivirus E^rns^, and apolipoprotein E (ApoE) were expressed in BHK-21 cells (Fig 6A) and infected with JEV NS1_I273H_. As we expected, although intracellular viral RNA replication and infectious titers in the culture supernatants of cells expressing either E^rns^ or ApoE were lower than those in the culture supernatants of cells expressing NS1, substantial viral replication and infectivity were observed in cells expressing E^rns^ or ApoE upon infection with JEV NS1_I273H_ at 96 hpi (Fig 6B). When we used a mutant ZIKV carrying the same mutation in NS1 (ZIKV NS1_I273H_), intracellular viral RNA replication and infectious titers in the culture supernatants were again observed in cells expressing either E^rns^, or ApoE (Fig 6C), suggesting that exchangeable apolipoproteins and E^rns^ play the same role as secretory NS1 in the infectious particle formation of flaviviruses. Moreover, either ApoE, wild type NS1, NS1_I273H_ or NS1_F160D_ was expressed in ApoE and ApoB double-knockout Huh7 cells and infected with hepatitis C virus (HCV), a member of the family *Flaviviridae* (Fig 6D). The levels of intracellular viral RNA replication were similar among the cells expressing either ApoE, wild type NS1, NS1_I273H_ or NS1_F160D_ upon HCV infection (Fig 6D, middle). However, the infectious titer in the culture supernatants and specific infectivity were impaired in cells expressing NS1_I273H_ (Fig 6D, left and right, respectively) at 72 hpi, suggesting that the secretory function of NS1 participates in the particle formation of HCV. These results suggest that secretory glycoproteins, including exchangeable apolipoproteins, pestivirus E^rns^, and flavivirus NS1, play a common role in the infectious particle formation of *Flaviviridae* viruses.

## Discussion

Flavivirus NS1 is a unique viral protein within the family *Flaviviridae* and is considered to be multifunctional; however, the role of NS1 in the virus life cycle has been less fully elucidated compared with the roles of other viral proteins, such as core, envelope, protease and polymerase. In this study, we investigated whether secretory NS1 plays a role in the virus particle formation of flaviviruses. By using *in silico* analysis of the protein-protein network and an *in vitro* complementation assay, we identified the amino acid residue involved in proper oligomerization for secretion and demonstrated that the lipid membrane-binding oligomer of NS1 is involved in the infectious particle formation. We also elucidated that the secretory glycoproteins commonly participate in the infectious particle formation of the *Flaviviridae* viruses.

In the early years of flavivirus research, NS1 was considered to participate in assembly and release of flavivirus particles due to its localization in the ER lumen and secretion profile [19]. Meanwhile, later studies using reverse genetics techniques to create deletion mutants of NS1 revealed that NS1 plays a crucial role in the formation of the replication complex through its interaction with NS4A and NS4B in the ER lumen [20, 21]. To further clarify the roles of NS1, in this study we tried to carry out the trans-complementation of NS1 by using DENV and JEV mutants lacking the NS1 gene. However, we were unable to rescue infectious viruses by the trans-complementation. Our attempt suggests that NS1plays crucial roles in the formation of the virus RNA replication complex that affects particle formation, and that complementation by the expression of NS1 depends on subtle structural differences in association with host and/or viral proteins [22]. Interestingly, by using the ZIKV carrying the dysfunctional NS1 by insertion of the HiBiT luciferase, infectious particles were successfully rescued by the exogenous expression of NS1 of other flaviviruses, suggesting that these different NS1 proteins can share the functional roles in the virus life cycle. In fact, NS1 is well conserved in flaviviruses and exhibits up to 80% amino acid similarity among them [3].

After translation, NS1 is considered to form a dimer and a higher oligomer including a putative hexamer. The dimer is located in the ER and is involved in RNA replication as previously reported [21, 23]. The secretory NS1 forms the higher oligomer [15]. Therefore, we hypothesized that the ER-resident dimer NS1 and the secretory NS1 play distinct roles in the virus life cycle. To investigate this hypothesis, we needed to identify the amino acid residues involved in the oligomerization of NS1; we therefore estimated the protein-protein interaction network within NS1 by using co-evolving amino-acid pairs [16, 17, 24]. We attempted to subject the data set of ZIKV to the co-evolution analysis, but the co-evolution value within ZIKV strains was too small to investigate further. We therefore employed sequences of the JEV serocomplex including JEV and WNV, and the data set shown in S1 Table was used for the *in vitro* analyses. Although the crystal structure of the dimer formation of NS1 was uncovered previously, the oligomerization of NS1 *in vitro* was conducted in an artificial manner [15, 25]. The co-evolving amino-acid pairs within NS1 were classified into 10 clusters, and the amino acid residue at position 273 was classified into cluster 8 (Fig 2A). The amino acid loci in cluster 8 are located on the β-ladder region which participates in neither lipid binding nor dimer formation [26]. In addition, FPLC analysis revealed that the NS1_I273H_ mutant was capable of forming the dimer but not a higher oligomer, suggesting that this computational analysis was useful for deducing co-evolving amino-acid pairs for estimation of the protein-protein interactions in a single viral protein.

**Fig 7:**
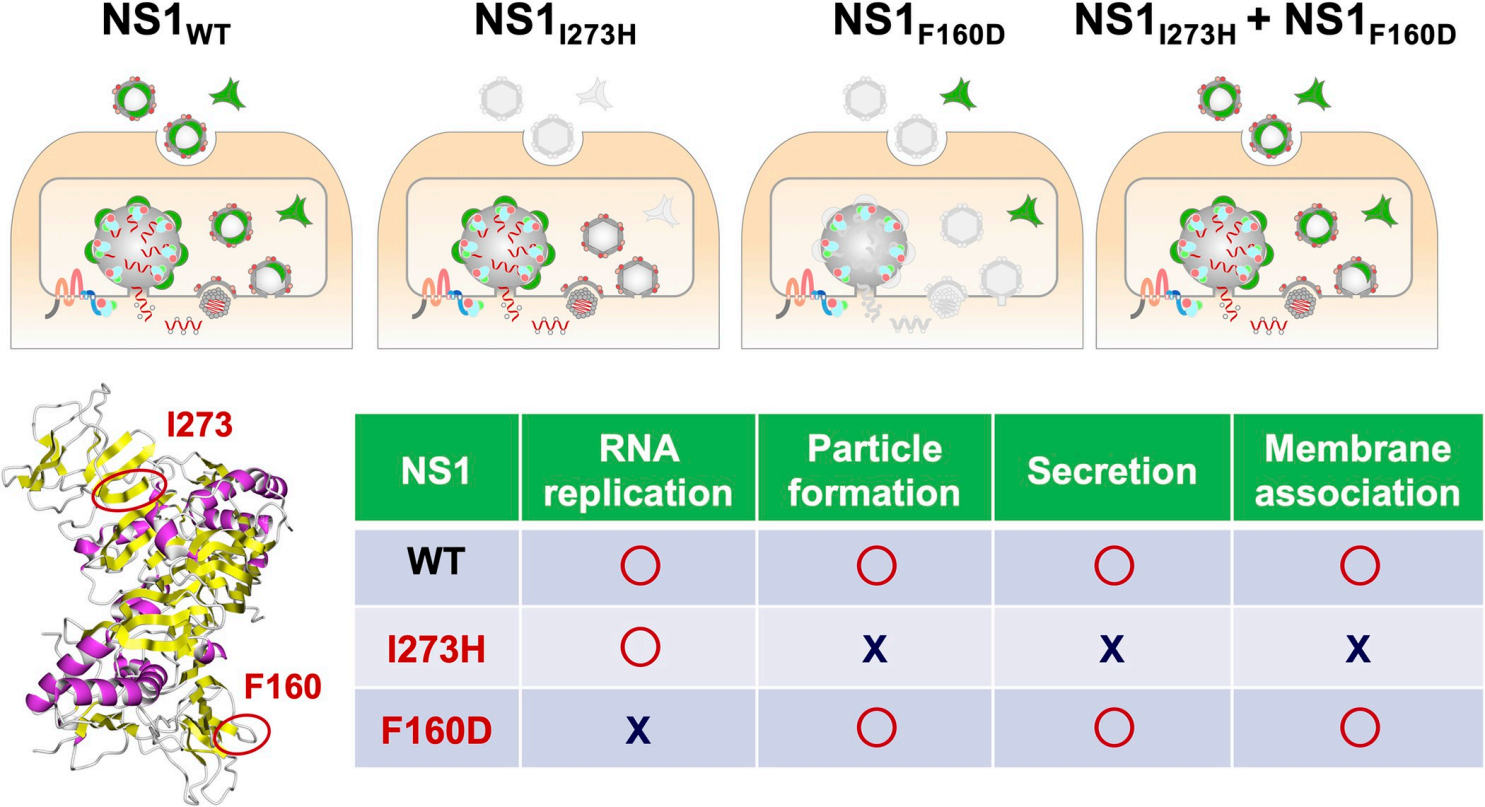
The secretory NS1 plays a crucial role in the infectious virus particle formation. The role of NS1 in viral RNA replication and the secretory machinery elucidated in this study are illustrated.

The previous study indicated that the secretory NS1 forms a lipoprotein particle with an open-barrel protein shell [25]. Thus, the formation of secretory NS1 is required for amphipathic properties and remodeling of the lipid membrane. Our previous studies have shown that amphipathic helices of exchangeable apolipoproteins play important roles in the infectious particle formation of hepaciviruses through their interaction with the viral membrane [11]. The amphipathic properties of viral secretory glycoproteins E^rns^ and NS1 participate in the infectious particle formation of hepaciviruses and pestiviruses [12]. In this study, we showed that JEV NS1_I273H_ retained its ability for RNA replication but lost its ability for infectious particle formation, and that NS1_I273H_ was incapable of being secreted into extracellular environments via the alteration of lipid membranes (second panel in Fig 7). These data suggest that the secretory property of NS1 is involved in the infectious particle formation. Future work to further investigate involvement of NS1 in later step of virus life cycle will be required. A study on DENV showed that mutations in NS1 impaired the production of infectious particles but had no effect on viral RNA replication [27]. On the other hand, Akey *et al*. showed that NS1 mutants incapable of generating replication complexes are nonetheless capable of remodeling the membranes of liposomes [15]. By exogenous expression of the NS1_F160D_ [15], production of the infectious particle of the JEV NS1_I273H_ was restored (Fig 4C). In accordance with these studies, we proved that NS1 possesses at least dual roles in the life cycle of flavivirus. In addition, we clearly showed that the exchangeable apolipoproteins and E^rns^ of pestivirus are capable of compensating for the particle-formation function lost in the NS1_I273H_ mutant. Furthermore, the expression of wild type NS1 and NS1_F160D_ but not of NS1_I273H_ was able to enhance the infectious particle formation of HCV in ApoB and ApoE double-knockout cells. These results indicate that the viruses belonging to the family *Flaviviridae* commonly utilize the amphipathic properties of secretory glycoproteins in the particle formation. To the best of our knowledge, our studies are the first to demonstrate the conversion of different viral proteins and host factors that share the same role and compensate for each other in the virus life cycle of the same virus family.

In summary, by means of computational analyses of the protein-protein network, evidence-based mutagenesis and physicochemical characterization, we have shown that the secretory NS1 protein participates in infectious particle formation (Fig 7). Further investigation of NS1 will be needed to clarify the role of this protein in the virus life cycle of *Flaviviridae* viruses.

## Materials and methods

### Plasmids

The cDNA clones of ApoE, pestivirus E^rns^, flavivirus NS1 from JEV, DENV-2, DENV-4 and ZIKV and AcGFP were inserted between the *Xho*I and *Xba*I sites of the lentiviral vector pCSII-EF-RfA [11] by using the Infusion technique, and the resulting plasmids were designated pCSII-EF-ApoE, pCSII-EF-E^rns^, pCSII-EF-NS1 and pCSII-EF-GFP, respectively. The mutant cDNAs of NS1 were amplified by PCR and inserted into pCSII-EF. The plasmids pMW119-DV4 [28], pMWJEAT [29] and rZKV(MR766-NIID)/pMV119 [30] encode full-length infectious clones of the DENV serotype 4 H241 strain (GenBank accession number: AY947539), JEV AT31 strain (GenBank accession number: AB196923) and ZIKV MR766 (GenBank accession number: LC002520), respectively. The cDNA clones which lack the NS1 gene, encode the HiBiT luciferase gene and carry mutations were constructed by using a KOD-Plus-Mutagenesis Kit (Toyobo) and the respective oligonucleotide primers. The plasmid pHH-JFH1 encodes a full-length cDNA of the JFH1 strain (GenBank accession number: AB047639) [31]. pHH-JFH1-E2p7NS2mt contains three adaptive mutations in pHH-JFH1. For obtaining recombinant baculoviruses, the cDNA clones of NS1 with an MBP tag were inserted into pFastBac vector (Thermo Fisher Scientific) with some modifications as previously described [32]. Then, the constructed plasmids were applied to a Bac-to-Bac system (Thermo Fisher Scientific) using DH10Bac to generate recombinant bacmids carrying the NS1 gene according to the manufacturer’s protocol. All of the plasmids used in this study were confirmed by sequencing with an ABI 3130 genetic analyzer (Thermo Fisher Scientific).

### Cell lines

All mammalian cell lines were cultured at 37°C under the conditions of a humidified atmosphere and 5% CO_2_. The human hepatocellular carcinoma-derived Huh7 cells, human embryonic kidney-derived 293T cells, African green monkey kidney-derived Vero E6 cells and baby hamster kidney fibroblasts-derived BHK-21 cells were maintained in DMEM (Nakalai) supplemented with 100 U/ml penicillin, 100 μg/ml streptomycin, and 10% fetal bovine serum. The Huh7-derived cell line Huh7.5.1 was kindly provided by Dr. F. Chisari. The Huh7 cells with double-knockout of apolipoproteins B and E (BE-KO cells) were established previously [11]. *Spodoptera frugiperda*-derived Sf-9 cells were maintained in Sf-900II insect medium (Thermo Fisher Scientific) supplemented with 100 U/ml penicillin and 100 μg/ml streptomycin and cultured at 28°C.

### Antibodies

Mouse monoclonal antibodies to β-actin, dsRNA and NS1 were obtained from MilliporeSigma, English & Scientific Consulting Kft and Bio Matrix research, respectively. Alexa Fluor (AF) 488-conjugated anti-rat IgG and 647-conjugated anti-mouse IgG antibodies were purchased from Thermo Fisher Scientific. Rat anti-HA and rabbit anti-NS1 antibodies were purchased from Roche Diagnostics and GeneTex, respectively. Rabbit anti-HCV NS5A was generated as described previously [33].

### Immunoblotting

Cells lysed on ice in lysis buffer (20 mM Tris-HCl [pH 7.4], 135 mM NaCl, 1% Triton-X 100, 10% glycerol) supplemented with a protease inhibitor cocktail, cOmplete mini (MilliporeSigma), were boiled in loading buffer and subjected to 5%–20% gradient SDS-PAGE. The proteins were transferred to polyvinylidene difluoride membranes (MilliporeSigma) and incubated with the appropriate antibodies. The immune complexes were visualized with SuperSignal West Femto substrate (Thermo Fisher Scientific). The signals were detected by use of an LAS-4000 image analyzer system (Fujifilm)/an Amersham Imager 600 (GE Healthcare) or an WSE-LuminoGraph I (ATTO)/ImageSaver6 (ATTO).

### Immunofluorescence

Cells were fixed with 4% paraformaldehyde (Nacalai Tesque). After fixation, the cells were permeabilized with PBS containing 0.2% Triton X-100 (Nacalai Tesque) for 10 min, blocked with 1% BSA fraction V (Millipore Sigma) in PBS, and then reacted with the indicated primary antibodies in PBS for 1 h at room temperature. After washing with PBS three times, cells were incubated with goat anti-mouse IgG Alexa Fluor 647–conjugated (1:1,500 dilution) or anti-rabbit IgG Alexa Fluor 488–conjugated (1:1,000 dilution) secondary antibodies in PBS for 1 h at room temperature. The cells were then counterstained with Hoechest 33342 (Dojindo) (1:2,000 dilution) for 10 min.

### Quantification of dsRNA production

For analysis of the production of dsRNA, confocal images were acquired with Eclipse Ti2 (Nikon) microscope, equipped with a PlanApo 20×/0.8 objective lens, a TI2-CTRE microscope controller (Nikon), a TI2-S-SE-E motorized stage (Nikon), an X-Light V3 (CrestOptics) spinning disk confocal unit, and a PRIME 95B scientific complementary metal-oxide semiconductor (sCMOS) camera (Teledyne Photometrics). The cells were illuminated with a CELESTA Light Engine laser light source (Lumencor) through a an FF01-391/477/549/639/741 excitation filter. Emission filters adopted for this experiment included an FF02-438/24 (Semrock) for Hoechst 33342, and an FF01-692/40 (Semrock) for Alexa 647. An FF421/491/567/659/776-Di01 dichroic mirror (Semrock) was used throughout this observation. Total fluorescent intensity within cells was quantified by using the ‘Multi Wavelength Cell Scoring’ module of MetaMorph software (Molecular Devices).

### Quantification of NS1 and dsRNA colocalization

Confocal images of the cells infected with ZIKV-HiBiT were acquired by IX83 (Olympus) microscope equipped with a UPlanSApo 60×/1.35 oil objective lens, a BioPoint MAC 6000 filter and shutter control unit (Ludl Electronic Products), an automated XY-stage (Chuo Precision Industrial), an X-Light V2 (CrestOptics) spinning disk confocal unit, and a PRIME BSI sCMOS camera (Teledyne Photometrics). The cells were illuminated with an LDI laser light source (Chroma Technology Corp.) through a ZET405/470/555/640x excitation filter (Chroma Technology Corp.). Emission filters adopted for these observations included ET440/40m for Hoechst 33342, BP510-550 (Olympus) for Alexa Fluor 488, and an FF01-692/40 (Semrock) for Alexa Fluor 647. A ZT405/488/555/640 dichroic mirror (Chroma Technology Corp.) was used in this observation. Colocalization area for the JEV NS1 (Alexa 488) and dsRNA (Alexa 647) was quantified with the use of the ‘measure colocalization’ function of MetaMorph software.

### Preparation of viruses

To obtain ZIKV and the mutants, BHK-21 cells were transfected with the cDNA clones by Trans IT LT-1 (Mirus), the culture supernatants were harvested, and infectious titers were determined by a focus-forming assay and expressed in focus-forming units (FFU). DENV, JEV and their mutants were generated as described previously [28]. In brief, the linearized infectious clones were transcribed by using an mMESSAGE mMACHINE T7 Ultra Kit, and the *in vitro*-transcribed RNA was electroporated. The supernatants were collected from the electroporated cells and infectious titers were determined by a focus-forming assay. pHH-JFH1-E2p7NS2mt was introduced into Huh7.5.1 cells, HCVcc in the supernatant was collected after serial passages, and infectious titers were determined by a focus-forming assay. All of the viruses were kept at -80°C until use. To generate the baculoviruses carrying the NS1, recombinant bacmids were transfected into Sf-9 cells by using X-tremeGENE HP DNA Transfection Reagent (MilliporeSigma). After incubating the cells for 4 days, the recombinant baculoviruses in the culture supernatants from the bacmid-transfected Sf-9 cells were collected and centrifuged at 1,500 *g* for 3 min to remove cells and debris. Then, the harvested supernatants were inoculated into naïve Sf-9 cells for 4 days. The passage 1 (P1) viruses were collected in a tube wrapped with foil and stored at 4°C until use.

### Purification of MBP-tagged NS1 and gel filtration

After inoculation of the recombinant baculoviruses P1 for 4 days in the Sf-9 cells, the cellular pellets were collected and resuspended with PBS containing EDTA and cOmplete mini. The cellular lysate was subjected to 2 freeze-thaw cycles and then centrifuged at 18,000 *g* for 10 min to remove cells and debris. The supernatants were transferred into Dextrin Sepharose High Performance (GE Healthcare) and purification of the MBP-tagged NS1 was performed according to the manufacturer’s protocol. Briefly, samples were poured into a column, washed with the binding buffer [20 mM TrisHCl (pH 7.4), 0.2 M NaCl, 1 mM EDTA and 1 mM DTT] and eluted with the elution buffer [0.5 M maltose, 20 mM TrisHCl (pH 7.4), 0.2 M NaCl, 1 mM EDTA and 1 mM DTT]. The size of the purified NS1 protein was examined by gel filtration using a Superose 6 column (Cytiva). Reaction was carried out at a flow rate of 0.5 ml/min with a buffer [50 mM phosphate buffer (pH 7.4) and 150 mM NaCl]. The loaded samples were fractionated into 39 fractions (500 μl each), and all fractions (20 μl) were analyzed by SDS-PAGE and immunoblotting using anti-NS1 antibody.

### Synthesis of liposome and interaction with NS1

Liposome solution was prepared as previously described [15]. The 1,2-Distearoyl-sn-glycero-3-phosphocholine (DSPC) and cholesterol (CHOL) were purchased from Avanti Polar Lipids and mixed at ratio of 1:9 CHOL:PC in chloroform. The lipid solutions were evaporated under a nitrogen gas stream to form a lipid film on the surface of glass tubes. Then, 400 μl of buffer [50 mM Bis-Tris (pH 5.5), 50 mM (NH_4_)_2_SO_4_, 10% glycerol] was added to the dried lipids and incubated in a water bath at 60°C for 10 min, followed by vortexing for 30 sec. Liposome solution (150 μl) and 150 μg of the purified NS1 protein were mixed and incubated for 2 h at 37°C with shaking. After centrifugation at 13,000 *g* for 20 min, the supernatant samples were subjected to negative staining.

### Negative staining and electron microscopy (EM)

The virus-infected cells were cultured on a Cell Desk polystyrene coverslip (Sumitomo Bakelite) and fixed with 4% paraformaldehyde at room temperature for 30 min. Cells were post-fixed for 1 h with 1% osmium tetroxide and 1% potassium ferrocyanide in 0.1 M sodium-phosphate buffer (pH 7.4), dehydrated in a graded series of ethanol and embedded in Epon812 (Taab Laboratory Equipment). 80 nm ultra-thin sections were stained with saturated uranyl acetate and lead citrate solution. Electron micrographs were obtained with a JEM-1400plus transmission electron microscope (JEOL). Liposomes containing the recombinant NS1 proteins were put on hydrophilic carbon-collodion grids and stained with 2% uranyl acetate. The grids were wick dried with filter paper, air dried for 10 min, and examined on a JEOL JEM-1400plus electron microscope at 80 kV. Images were captured on an Olympus Veleta 2K x 2K side-mounted TEM CCD camera.

### Luciferase assay

Luciferase activity was measured by using a Bright-Glo luciferase assay system (Promega) and Nano-Glo HiBiT Lytic assay system (Promega) according to the protocol provided by the manufacturer.

### Quantitative RT-PCR

For quantification of viral RNA copies, total RNA was extracted from cells by using a PureLink RNA Mini Kit (Thermo Fisher Scientific), and then first-strand cDNA synthesis and qRT-PCR were performed by using a TaqMan RNA-to-C_T_ 1-step Kit and ViiA7 system (Thermo Fisher Scientific), respectively, according to the manufacturer’s protocols. For quantification of viral RNA, the primer sets for the detection of the noncoding region reported in the previous studies [34–36] were used. Fluorescent signals were determined by the ViiA7 system.

### Computational intra-protein network analysis

The intra-protein interaction was analyzed by the BIS. The methodology and related co-evolution signal analysis are described in previous studies [16, 17].

### Statistical analysis

All assays were performed in triplicate and independently repeated at least two times. Results were expressed as the means±standard deviations or standard errors. Statistical significance was determined by the two-tailed Student’s *t*-test, one-way ANOVA with Dunnett’s test, or Kruskal–Wallis test performed by GraphPad Prism (Software ver. 9.2.0). Significantly different values (*p*<0.05) are indicated by an asterisk.

## Supporting information

S1 Fig Trans-complementation of NS1 is disabled by using cDNA clones possessing deletion in the NS1 genes of JEV or DENV. (A) An illustration shows the experimental workflow. A schematic representation of the deletion mutants of flaviviruses. The region of the deletion was 12–894 nt (4–298 aa) of NS1. *In vitro*-transcribed RNAs of the NS1 deletion mutants were transfected into Vero E6 cells expressing NS1, and infectious titers in the culture supernatants were determined upon transfection into Vero E6 cells. Expression of JEV (B) or DENV (C) NS1 was determined by immunoblotting at 48 hpi of lentiviruses into Vero E6 cells. Infectious titers in the culture supernatants were determined upon either transfection of the *in vitro*-transcribed RNA of the NS1 deletion mutants or infection of wild type viruses at 4 days post-transfection or infection.
(TIFF)

S2 Fig The profile of the NS1 mutant screening. Expression of the respective NS1 was determined by immunoblotting at 48 hpi of lentiviruses into BHK-21 cells. (B) The production of double-stranded RNA (dsRNA) was determined by staining with antibody against dsRNA. The signal of staining was quantified and calculated (See Materials and Methods) and shown in a dot graph. The red color indicates positive of dsRNA.
(TIFF)

S3 Fig NS1 does not involve in viral translation. (A) A schematic representation of the DENV replicon carrying the cassette of the secreted NanoLuc luciferase gene within the C gene. The “GAA” in NS5 gene indicates the replication-defective mutant. (B) An illustration shows the experimental workflow. Expression of the NS1 proteins (wildtype, F160D, and I273H) was determined by immunoblotting at 48 hpi of lentiviruses into Huh7 cells. (C) Luciferase activity in supernatants of the cells were determined at 4- and 24-h post transfection of the wildtype and the replication-defective “GAA” replicons.
(TIFF)

S1 Table Coevolution network of the Japanese encephalitis serocomplex group. Full-length of NS1 (352 amino acids) sequences of the 67 strains of Japanese encephalitis serocomplex including JEV and WNV were aligned and 10 clusters were identified by BIS. For each cluster, the positions of the different coevolving residues or blocks and corresponding *p*-value, are indicated.
(XLSX)

S2 Table Amino acid substitutions of NS1 used for the screening. The 50 positions within NS1 were substituted for generation of mutant NS1. Lentiviruses of the indicated mutant NS1 were subjected to the screening.
(XLSX)
