# Supplementary figures and images for: Secretory glycoprotein NS1 plays a crucial role in the particle formation of flaviviruses
Source: PLoS Pathog. 2022 Jun 3;18(6):e1010593. doi: 10.1371/journal.ppat.1010593 (PMC9200304; doi:10.1371/journal.ppat.1010593)

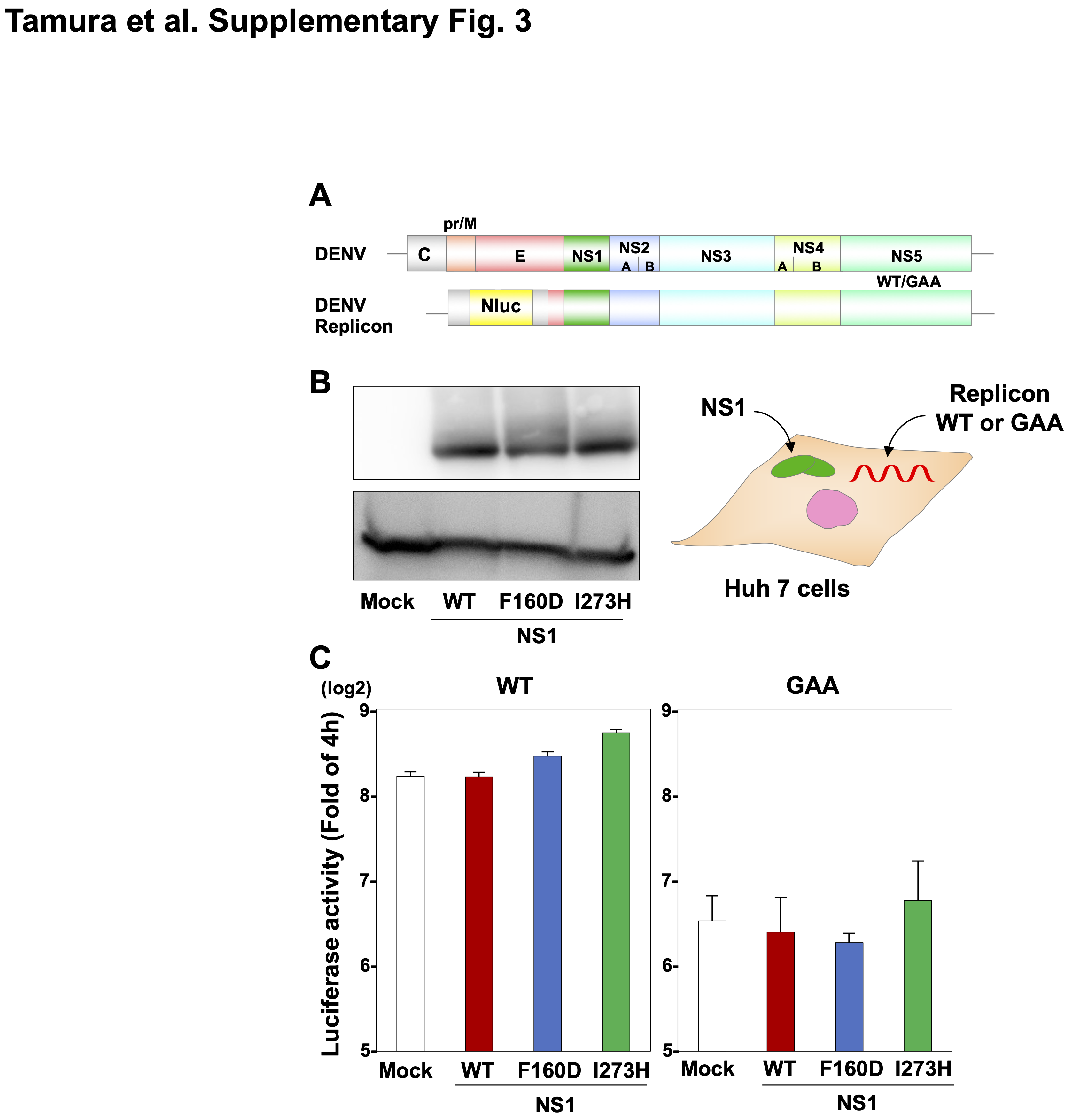

Supplement: S3 Fig — (A) A schematic representation of the DENV replicon carrying the cassette of the secreted NanoLuc luciferase gene within the C gene. The “GAA” in NS5 gene indicates the replication-defective mutant. (B) An illustration shows the experimental workflow. Expression of the NS1 proteins (wildtype, F160D, and I273H) was determined by immunoblotting at 48 hpi of lentiviruses into Huh7 cells. (C) Luciferase activity in supernatants of the cells were determined at 4- and 24-h post transfection of the wildtype and the replication-defective “GAA” replicons. (TIFF) [file ppat.1010593.s003.tiff]
